# Effects of the Antimicrobial Peptide LL-37 and Innate Effector Mechanisms in Colistin-Resistant *Klebsiella pneumoniae* With *mgrB* Insertions

**DOI:** 10.3389/fmicb.2019.02632

**Published:** 2019-11-14

**Authors:** Hissa M. Al-Farsi, Salma Al-Adwani, Sultan Ahmed, Carmen Vogt, Anoop T. Ambikan, Anna Leber, Amina Al-Jardani, Saleh Al-Azri, Zakariya Al-Muharmi, Muhammet S. Toprak, Christian G. Giske, Peter Bergman

**Affiliations:** ^1^ Department of Laboratory Medicine, Division of Clinical Microbiology, Karolinska Institutet, Stockholm, Sweden; ^2^ Central Public Health Laboratories, Ministry of Health, Muscat, Oman; ^3^ Department of Animal and Veterinary Sciences, College of Agricultural and Marine Sciences, Sultan Qaboos University, Muscat, Oman; ^4^ Department of Applied Physics, Biomedical and X-Ray Physics, KTH Royal Institute of Technology/AlbaNova, Stockholm, Sweden; ^5^ Department of Microbiology & Immunology, College of Medicine & Health Sciences, Sultan Qaboos University, Muscat, Oman; ^6^Department of Clinical Microbiology, Karolinska University Hospital, Stockholm, Sweden; ^7^ Infectious Disease Clinic, The Immunodeficiency Unit, Karolinska University Hospital, Stockholm, Sweden

**Keywords:** cross-resistance, colistin, LL-37, innate immunity, zeta potential, whole blood killing assay, serum killing assay, zebrafish

## Abstract

**Background:**

Colistin is a polypeptide antibiotic drug that targets lipopolysaccharides in the outer membrane of Gram-negative bacteria. Inactivation of the *mgrB*-gene is a common mechanism behind colistin-resistance in *Klebsiella pneumoniae* (Kpn). Since colistin is a cyclic polypeptide, it may exhibit cross-resistance with the antimicrobial peptide LL-37, and with other innate effector mechanisms, but previous results are inconclusive.

**Objective:**

To study potential cross-resistance between colistin and LL-37, as well as with other innate effector mechanisms, and to compare virulence of colistin-resistant and susceptible Kpn strains.

**Materials/Methods:**

Carbapenemase-producing Kpn from Oman (*n* = 17) were subjected to antimicrobial susceptibility testing and whole genome sequencing. Susceptibility to colistin and LL-37 was studied. The surface charge was determined by zeta-potential measurements and the morphology of treated bacteria was analyzed with electron microscopy. Bacterial survival was assessed in human whole blood and serum, as well as in a zebrafish infection-model.

**Results:**

Genome-analysis revealed insertion-sequences in the *mgrB* gene, as a cause of colistin resistance in 8/17 isolates. Colistin-resistant (Col-R) isolates were found to be more resistant to LL-37 compared to colistin-susceptible (Col-S) isolates, but only at concentrations ≥50 μg/ml. There was no significant difference in surface charge between the isolates. The morphological changes were similar in both Col-R and Col-S isolates after exposure to LL-37. Finally, no survival difference between the Col-R and Col-S isolates was observed in whole blood or serum, or in zebrafish embryos.

**Conclusion:**

Cross-resistance between colistin and LL-37 was observed at elevated concentrations of LL-37. However, Col-R and Col-S isolates exhibited similar survival in serum and whole blood, and in a zebrafish infection-model, suggesting that cross-resistance most likely play a limited role during physiological conditions. However, it cannot be ruled out that the observed cross-resistance could be relevant in conditions where LL-37 levels reach high concentrations, such as during infection or inflammation.

## Introduction

*Klebsiella pneumoniae* (Kpn) is a significant nosocomial pathogen worldwide. It causes an array of infections including bloodstream infections, urinary tract infection, pneumonia, peritonitis, and occasionally hospital-acquired meningitis (Paczosa and Mecsas, 2016). Carbapenemase-producing Kpn is a major threat in the clinical setting due to the limited number of treatment options. Many clinical isolates are only susceptible to colistin (polymyxin E), which therefore has emerged as the last treatment resort. However, the increasing use of colistin is mirrored with an increasing bacterial resistance against this drug globally, including Oman in the Arabian Peninsula (Liu et al., 2016; Sonnevend et al., 2016; Mohsin et al., 2018).

Colistin is an antimicrobial peptide-related compound, with a net positive charge at physiological pH. It binds to negatively charged phosphate groups in the lipid A component of lipopolysaccharides (LPS). As a consequence, the binding leads to disruption and loss of bacterial cell membrane integrity, causing cell death (Giske, 2015). Resistance to colistin is assumed to be caused by reducing the net negative charge of lipid A. The charged state of the bacterial surface can be assessed by measuring the zeta potential of the bacterial surface (Fukuoka et al., 2008).

Colistin-resistance in Gram-negative bacteria can be mediated via the plasmid-associated *mcr* genes (Sun et al., 2018). Interestingly, *mcr-1* can be targeted with an inhibitor that can restore antibiotic susceptibility to colistin in carbapenem-resistant Enterobacteriaceae (Zhou et al., 2019). In addition, colistin-resistance in Kpn is commonly caused by alterations in the *mgrB* gene, which encodes a negative-feedback regulator of the PhoQ-PhoP signaling system, resulting in the upregulation of the Pmr lipopolysaccharide modification system (Cannatelli et al., 2014).

Colistin and the antimicrobial peptide LL-37 share similar bacteria-binding mechanisms, leading to the unresolved hypothesis that cross-resistance between colistin and LL-37 exists. Some studies have supported the cross-resistance hypothesis (Llobet et al., 2009; Napier et al., 2013; Kadar et al., 2015), whereas others have shown contrasting results (Moffatt et al., 2013) or no correlation (Garcia-Quintanilla et al., 2014; Dobias et al., 2017). In addition, altered virulence of Kpn as a result of colistin-resistance is also a matter of debate and some studies showed unaltered virulence by *mgrB*-insertions (Cannatelli et al., 2015; Arena et al., 2016), whereas another study reported increased virulence (Kidd et al., 2017).

Bacterial virulence can be assessed by several different *in vitro* and *in vivo* methods. Common *in vitro* systems include whole blood and serum bactericidal assays. The former assesses the combined effects of complement activity, opsonization, phagocytosis and intracellular killing, while the serum bactericidal assay mainly evaluates bacterial susceptibility to complement activity. *In vivo* models are often performed in mice but more simple animal-models can be used. The zebrafish model has increasingly been used to evaluate innate immune mechanisms, including Kpn-virulence (Marcoleta et al., 2018). Many innate immune pathways are conserved between mammals and zebrafish, including complement, antimicrobial peptides and phagocytic host defenses, here collectively designated as “innate effector mechanisms” (Marcoleta et al., 2018).

We investigated potential cross-resistance between colistin and innate effector mechanisms using a clinical collection of Col-R and Col-S isolates from Oman. Strains were examined with antimicrobial susceptibility testing, whole genome sequencing, and electron microscopy imaging, and studies on bacterial survival were conducted in whole blood and serum, as well as in a zebrafish infection-model.

## Materials and Methods

### Reagents

Blood and CLED agar plates, phosphate buffered saline (PBS; 10 mM; pH 7.4), lysogeny broth (LB; pH 7.5), cation-adjusted Muller-Hinton Broth (CaMHB), and sterilized deionized water (WID; pH 7.0) were obtained from the Substrate Unit at Karolinska University Hospital, Stockholm, Sweden. RPMI-1640 was purchased from Invitrogen. Sodium polyanethole sulfonate (SPS) and colistin sulfate were purchased from Sigma-Aldrich. Stock solutions were prepared in PBS at 1000 μg/ml for colistin and 10 mg/ml in sterilized distilled water for SPS. LL-37 was purchased from Innovagen (Lund, Sweden) and dissolved in 0.1% trifluoroacetic acid (TFA; Fluka BioChemika), aliquoted and stored at −20°C until used. The E3 medium (5 mM NaCl, 0.17 mM KCl, 0.33 mM CaCl_2_, and 0.33 mM MgSO_4_) was provided by the zebrafish core facility at Karolinska Institutet, Stockholm, Sweden.

### Bacterial Strains

As a national health policy in Oman, all clinical samples with decreased susceptibility to carbapenem are sent to a central public health laboratory for further characterization. Out of 245 Kpn isolates with preliminary carbapenem resistance, 8 showed reduced susceptibility to colistin due to *mgrB*-insertion and were included in this study. Additionally, 9 isolates were selected from the collection using a random number table and included in the study as a control group. All isolates included in this study were collected between January and August 2015. Carbapenem susceptibility was assessed by disk diffusion, and colistin susceptibility was assessed by broth micro-dilution (BMD), according to the EUCAST guidelines (The European Committee on Antimicrobial Susceptibility Testing [EUCAST], 2016). Two genetically similar strains from this collection were studied further, Col-S (OM322) and Col-R (OM124). Additional strains were used as controls: *K. pneumoniae* (ATCC25955), *E. coli* D-21 strain (CGSC5158) and *Proteus mirabilis* (ATCC29245).

### Whole Genome and Sanger Sequencing

All isolates (*n* = 17) were subjected to DNA extraction using the MagNA Pure 96 System (Roche Diagnostics Nederland, Almere, Netherlands). Whole genome sequencing (WGS) was performed for these isolates using HiSeq 2500 (Illumina, San Diego, CA, United States) at SciLifeLab (Solna, Sweden). Raw reads data generated in this study were deposited in SRA as project PRJNA544438. The reads were assembled into contigs using SPAdes (ver 3.9.0) (Bankevich et al., 2012). Sequence types (ST) and core genome MLST were assigned according to the Kpn MLST database (Jolley and Maiden, 2010). The same database was used to identify virulence genes whereas capsular type was determined using Kaptive web (Wick et al., 2018). Resistance genes were identified using ResFinder (Zankari et al., 2012) and CARD (McArthur et al., 2013). *In silico* DNA-DNA hybridization (DDH) using the generalized linear model (GLM) and formula 2, as well as average nucleotide identity (ANI) analysis, were employed for selected strains as described previously (Meier-Kolthoff et al., 2013; Rodriguez-R and Konstantinidis, 2016). SNP calling was done for selected strains with Microbial Genomics Module 3.0 of CLC Genomics Workbench 11.0 (QIAGEN bioinformatics, Aarhus, Denmark). PCR for the *mgrB* gene was performed as described elsewhere (Cannatelli et al., 2014) and Sanger sequencing was done to capture the sequence of the insertion element (IS) and identify its type using the ISfinder database (Siguier et al., 2006).

### Bacterial Killing Assays for the Genetically Similar Col-R and Col-S Strains

Bacteria were grown in CaMHB at 37°C with shaking (220 rpm) to reach exponential phase (OD_600__nm_ = 0.4–0.6), and were diluted to the starting bacterial inoculum in CaMHB or LB media prior to each experiment. Bacteria were incubated with either LL-37, blood or serum. After 2 h of incubation, bacteria were serially diluted then plated on blood agar and incubated overnight to measure colony forming unit (CFU) counts. For the LL-37-killing assay, the bacterial inoculum (∼5 × 10^7^ CFU/ml) was treated with a range of LL-37 concentrations (3–100 μg/ml) in CaMHB. For the blood killing assay, 100 μl of the bacterial inoculum (∼5 × 10^7^ CFU/ml) was incubated with 900 μl of 40% whole blood in RPMI-1640 medium. For the serum killing assay, complement components in pooled sera was either heat inactivated (56°C, 30 min) or chemically inactivated by incubation with 1 mg/ml of SPS for 30 min at room temperature (Palarasah et al., 2010). Treated and non-treated sera diluted to 20% in PBS (900 μl) was incubated with 100 μl of each overnight bacterial culture (Col-R, Col-S, ATCC25955, *E. coli* D-21). For each strain in each experiment, bacteria without treatment and media alone were used as growth controls and negative controls, respectively. Each assay was performed at least in three independent experiments.

### Growth Curve Measurements

Bacterial growth was measured in the BioScreen C MBR instrument (Oy Growth Curves AB Ltd.) which allows turbidity measurements at an optical density (OD_600__nm_) in a precision incubator (37°C) equipped with a linear shaker. An initial bacterial inoculum corresponding to an OD_600_ of 0.05 (1 × 10^3^ CFU/ml) in logarithmic phase from each Kpn strain (ATCC25955, Col-S, Col-R) and *P. mirabilis* (ATCC29245) was incubated with 100 μg/ml of LL-37. Measurements were made every 15 min over a time period of 20 h. OD_600_ curves were generated by plotting turbidity versus time. Each sample was analyzed in duplicate.

### Measurement of Bacterial Zeta Potential

The zeta potential of the bacteria was measured at 25°C using the Zetasizer Nano ZS90 (Malvern Instruments, United Kingdom) instrument as described previously (Soon et al., 2011). Measurements were performed in two different diluents; sterile deionized water (DIW; pH 7.0) and PBS (10 mM; pH 7.4) in triplicates on three independently prepared bacterial suspensions for Kpn strains (Col-R, Col-S, ATCC25955). Bacteria were grown to exponential growth phase as described previously. A stock suspension of 1 × 10^9^ CFU/ml was prepared by harvesting the cells from the broth culture by three centrifugation cycles (3000 × *g* for 10 min at 25°C) and suspended in DIW or PBS. A 10-fold dilution from the stock suspension was performed immediately prior to each measurement. The surface charge analysis was performed using electrophoretic mobility measurements and the Helmholtz–Smoluchowski equation was used for zeta potential calculations.

### Electron Microscopy

For transmission electron microscope (TEM) and scanning electron microscope (SEM) analysis, the two genetically similar strains (Col-S and Col-R) were grown to log phase and diluted to ∼1 × 10^8^ CFU/ml in CaMHB. Bacteria were left untreated as control or were incubated with LL-37 at different concentrations (50–200 mg/ml) at 37°C for 0.5 h or 2 h. Then, CFU counts were performed in parallel. For TEM, mixtures were fixed in 2.5% glutaraldehyde in PBS (0.1 M, pH 7.4) at room temperature for 30 min and rinsed in PBS prior to post-fixation using 2% osmium tetroxide in PBS at 4°C for 2 h. The samples were subsequently dehydrated in ethanol followed by acetone and finally embedded in LX-112. Ultrathin sections were prepared using a Leica EM UC7 (Leica Microsystems) and contrasted with uranyl acetate followed by Reynolds lead citrate. The sections were examined in a Tecnai Spirit G2 Bio TWIN Electron microscope (Tecnai High-Technologies) at 100 kV, and images acquired using a 2k × 2k Veleta CCD camera (Olympus Soft Imaging Solutions GmbH). SEM was performed to compare changes in the bacterial surface between non-exposed bacteria and those exposed to a range of LL-37 concentrations (50–200 μg/ml). For SEM, samples were fixed using 2.5% glutaraldehyde PBS. The fixed samples were adhered onto a pore membrane and washed in Milli-Q water prior to stepwise ethanol dehydration and critical-point-drying using carbon dioxide (Leica EM CPD 030). The pore membranes were mounted on specimen stubs using carbon adhesive tabs and sputter coated with platinum (Quorum Q150T ES). SEM images were acquired using an Ultra 55 field emission scanning electron microscope (Zeiss, Oberkochen, Germany) at 5 kV and the SE2 detector. Both TEM and SEM analyses were performed three times for each strain.

### Zebrafish Infection Model

The fertilized zebrafish embryos were lined against a glass slide in a petri dish and microinjection was performed using a glass needle (Harvard apparatus, Quebec, Canada) controlled with a micromanipulator Narishige MN-153 (Narishige International Limited, London, United Kingdom) connected to an Eppendorf FemtoJet express (Eppendorf AG, Hamburg, Germany). Microinjection (1–2 nL) of the bacterial suspension (approximately 150–300 CFU of bacteria in E3 medium) was performed into the yolk sac in 4–5 h post fertilized embryos. In parallel, a control group was injected with E3 medium only. To determine the number of bacteria in the injected volume, one drop was spread on agar plates before injection. To determine the bacterial counts in the embryos, 1–3 embryos were digested and plated just immediately after injection. Injected embryos were transferred into a petri dish with E3 medium and incubated at 30°C for 72 h and observed for any sign of disease and survival twice a day under a stereomicroscope. Both experimental settings were repeated four times.

### Statistical Analysis

Data were analyzed by GraphPad Prism version 8.0.1 (GraphPad software). Normality was tested using the Shapiro-Wilk test. Based on normality test and number of groups examined parametric tests (unpaired student’s *t* test or ANOVA) or non-parametric tests (Mann-Whitney or Kruskal–Wallis tests) were used. If the *P* value for multi-group comparisons (ANOVA and Kruskal–Wallis tests) were significant, *post hoc* tests were performed using Tukey’s test (parametric data) or Dunn’s test (non-parametric data) to obtain *P* values for specific comparisons of interest between group means or medium. A *P* value of <0.05 was used for statistical significance. Log-rank (Mantel-Cox) test was used for survival analyses.

## Results

### Inactivation of *mgrB* Is a Common Mechanism of Colistin-Resistance in Kpn From Oman

The 17 clinical Kpn isolates were selected by virtue of being carbapenem resistant (Table 1). Notably, the majority of isolates produced the metallo-β-lactamase NDM-1 or different OXA-48-like enzymes, which caused carbapenem resistance (Table 1). In addition to carbapenem resistance, 8 out of the 17 isolates were Col-R. They showed wild type *PmrA/PmrB* and *PhoP/PhoQ* genes. However, the characterization of *mgrB* mutations by sequencing revealed the presence of insertion elements (IS) in the Col-R strains, with some diversity in IS types and site of insertion. Most strains (*n* = 6/8) had IS*Kpn14* at different sites. One isolate harbored IS*Kpn25* and another had an IS*Kpn14*-like insertion element.

**TABLE 1:** Description of the isolates.

	**ID**	**Hospital**	**Source**	**Colistin treatment**	**β-lactam type^∗^**	**ST**	**Mer MIC (μg/ml)**	**Colistin^∗∗^ MIC (μg/ml)**	***MgrB* (nt)^∗∗∗^**	**Capsule**	**O-serotype**	**Virulence genes**
Colistin resistant strains	OM124	Khoula	Urine	Yes	NDM-1	11	11	16	IS*Kpn14* (37)	KL14	O3b	*acrABR, mrkABCDFHLJ, ureABD*
	OM501	Khoula	Wound	No	OXA-48	101	12	8	IS*Kpn14*-like (102)	KL17	O1v1	*acrABR, irp1, irp2, kfuABC, mrkABCDFHLJ, ureABD*
	OM536	Nizwa	Urine	NA	NDM-1	11	12	16	IS*Kpn14* (37)	KL14	O3b	*acrABR, mrkABCDFHLJ, ureABD*
	OM568	Khoula	Wound	No	NDM-1 + OXA-48	101	9	16	IS*Kpn14* (102)	KL17	O1v1	*acrABR, irp1, irp2, kfuABC, mrkABCDFHLJ, ureABD*
	OM588	Khoula	Fecal	No	OXA-48	101	10	16	IS*Kpn14* (37)	KL17	O1v1	*acrABR, irp1, irp2, kfuABC, mrkABCDFHLJ, ureABD*
	OM1234	Nizwa	Urine	No	OXA-232	231	11	8	IS*Kpn25* (23)	KL51	O1v2	*acrABR, irp1, irp2, iucABCD, iutA, kfuABC, mrkABCDFHLJ, ureABD*
	OM300	Nizwa	NA	NA	NDM-1	11	NA	32	IS*Kpn14* (37)	KL14	O3b	*acrABR, mrkABCDFHLJ, ureABD*
	OM290	Nizwa	Urine	No	NDM-1	11	12	32	IS*Kpn14* (37)	KL14	O3b	*acrABR, mrkABCDFHLJ, ureABD*
Colistin Susceptible strains	OM322	Nizwa	Urine	No	NDM-1	11	11	<1	NA	KL14	OL104	*acrABR, mrkABCDFHLJ, ureABD*
	OM003	SQH	Fecal	No	OXA-232	231	30	<2	NA	KL15	O1v2	*acrABR, irp1, irp2, kfuABC, mrkABCDFHLJ, ureABD*
	OM193	SQH	respiratory	No	CTX-M-15	11	21	<2	NA	KL15	O4	*acrABR, irp1, irp2, mrkABCDFHLJ, ureABD*
	OM334	Nizwa	NA	NA	OXA-232	231	11	<2	NA	KL51	O1v2	*acrABR, irp1, irp2, kfuABC, mrkABCDFHLJ, ureABD*
	OM442	SQH	Urine	Yes	NDM-1	147	14	<2	NA	KL64	O2v1	*acrABR, irp1, irp2, mrkABCDFHLJ, ureABD*
	OM562	Khoula	Fecal	No	NDM-1	15	15	<2	NA	KL24	O1v1	*acrABR, irp1, irp2, kfuABC, mrkABCDFHLJ, ureABD*
	OM589	Khoula	Biopsy	Yes	OXA-181	147	24	<2	NA	KL64	O2v1	*acrABR, irp1, irp2, mrkABCDFHLJ, ureABD*
	OM876	NA	NA	NA	NDM-1	11	7	<2	NA	KL14	O3b	*acrABR, mrkABCDFJ, ureABD*
	OM1794	Ibri	Respiratory	No	NDM-1	11	12	<2	NA	KL15	O4	*acrABR, irp1, irp2, mrkABC, ureABD*
	ATCC25955					1271			NA	KL28	O1v2	*acrABR, ureABD*

*^∗^All clinical isolates carried *bla*_*CTX*–*M*–*15*_. ^∗∗^Broth microdilution assay was used for measuring minimum inhibitory concentration values. ^∗∗∗^All colistin resistance strains showed intact *PmrA/PmrB* and *PhoP/PhoQ* genes. NA: not determined. MIC: minimum inhibitory concentration. nt: nucleotides. mer: Meropenem.*

Interestingly, this collection contained two Kpn urine isolates with similar antibiotic susceptibility patterns against a large panel of antibiotics (Supplementary Figure S1) but one isolate OM124 was colistin resistant (Col-R, MIC 16 μg/ml) and the other, OM322, was colistin susceptible (Col-S, MIC < 1 μg/ml). Sanger-sequencing revealed that there was a 777 bp insertion at nucleotide 37 of the *mgrB* gene in the colistin-resistant isolate (OM124). This insertion, which was absent in OM322, was a likely explanation of the colistin-resistant phenotype in isolate OM124 (Supplementary Figure S1). It was identified as IS*Kpn14*, a part of the IS1 Superfamily.

The strains had very close genetic relationship. They shared identical virulence genes and capsule types but had different O-serotypes. All 8 genes that are used to define the O-serotype in the Kaptive database (*manC*, *manB*, *wzm*, *wzt*, *wbdD*, *wbdA*, *wbdB*, and *wbdC*) were present in OM124, whereas in OM322 *mantB* was absent and only seven genes were detected. However, both shared an identical core genome of 694 genes and belonged to ST11 (CG258) (Table 1). *In silico* DNA-DNA hybridization (DDH) showed that the isolates were identical to 99.9%. They differed only by 0.03% in GC content and by only 14 SNPs (0.3%) out of 4719 compared SNPs after filtering. Also, their average nucleotide identity (ANI) was 100%. The very close genetic relationship between these strains made them suitable for experiments to dissect the role of *mgrB* and colistin-resistance in relation to innate effector mechanisms.

### Cross-Resistance Between Colistin and LL-37 Is Only Observed at LL-37 Concentrations ≥50 μg/ml

**FIGURE 1:**
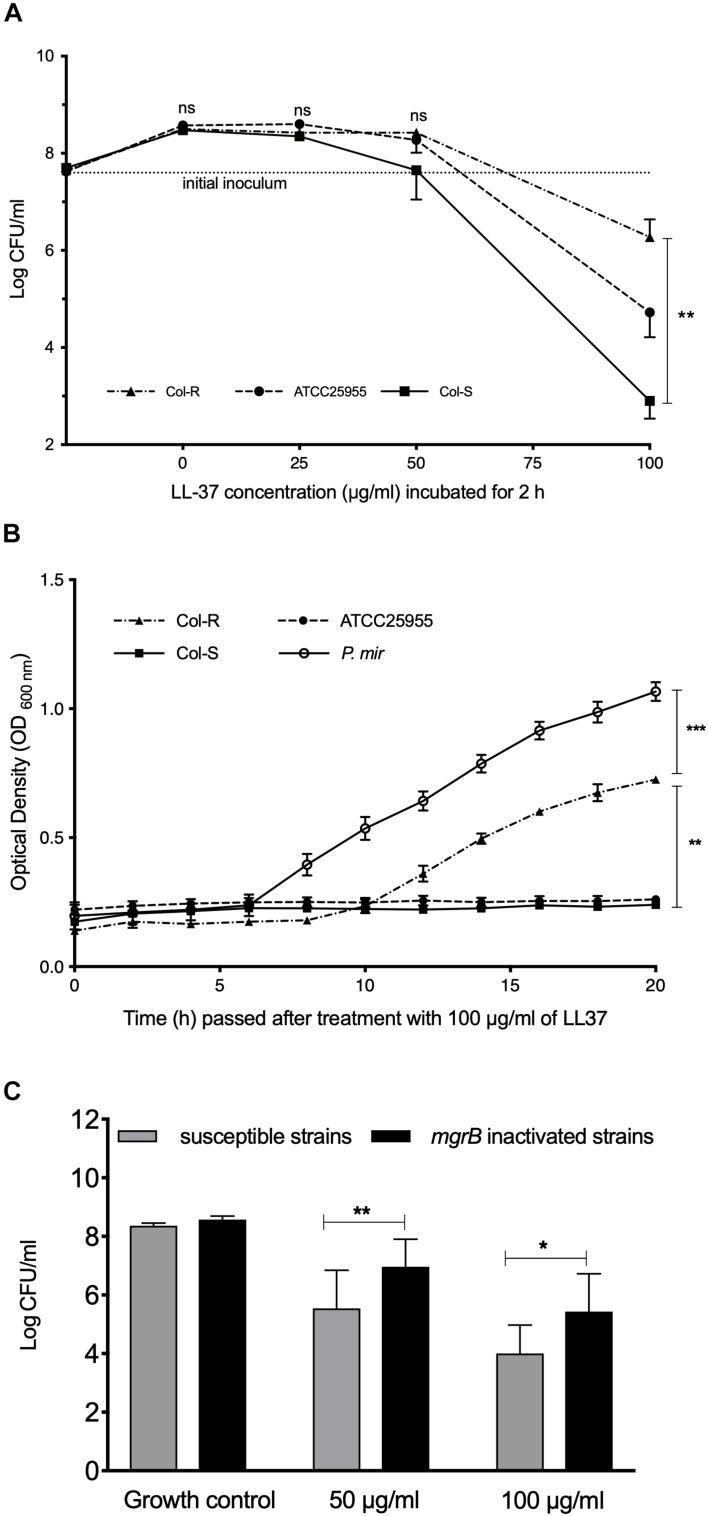
The effect of LL-37 on colistin-resistant and colistin-susceptible strains. **(A)** Bacterial Intial incoulom (dotted line) was exposed to a range of LL-37 concentrations (3–100 μg/ml) for 2 h. The non-parametric Kruskal–Wallis test was used for statistical analysis. **(B)** Growth curves were measured using Bioscreen C. Bacteria (∼1 × 10^3^ CFU/ml) were incubated with 100 μg/ml of LL-37 for 20 h. One-way ANOVA was used for statistical analysis. **(C)** Initial bacterial inoculum (∼5 × 10^7^ CFU/ml) of Col-S isolates (*n* = 9) and Col-R isolates (*n* = 8) with *mgrB* gene insertion elements were exposed to 50 μg/ml or 100 μg/ml of LL-37 for 2 h. One way ANOVA was used for statistical analysis. In all assays **(A–C)**, the lowest limit of detection was (∼1 × 10^2^ CFU/ml). Data plotted are mean ± SEM. Bacteria were grown in CaMHB media. ^∗^*P* ≤ 0.05; ^∗∗^*P* ≤ 0.01; ^∗∗∗^*P* ≤ 0.001.

The three Kpn isolates (ATCC25955, Col-S and Col-R) were incubated with different concentrations of LL-37 (Figure 1A). Notably, no obvious effect of LL-37 against any of the isolates at concentrations below 50 μg/ml was observed. At 50 μg/ml, the Col-S strain appeared to be more susceptible to LL-37 than the Col-R strain, but the difference did not reach statistical significance. However, at 100 μg/ml there was a profound difference between the strains with regard to susceptibility to LL-37 (*p* = 0.0098). The results from the BioScreen-experiments verified this finding and revealed that 100 μg/ml of LL-37 inhibited all growth of the Col-S strain, whereas the Col-R strain exhibited almost normal growth (*p* = 0.0041). As a control of the experimental system, the intrinsically colistin-resistant *P. mirabilis* was examined in parallel in the BioScreen-system and was completely resistant to LL-37 (Figure 1B). Next, we exposed all strains in the collection with *mgrB*-insertions (*n* = 7) and all colistin susceptible strains (*n* = 9) to 50 and 100 μg/ml of LL-37 (Table 1). The cross-resistance with LL-37 was statistically significant at 50 μg/ml (*P* = 0.0078) and at 100 μg/ml (*P* = 0.028) (Figure 1C).

### The *mgrB*-Insertion Does Not Alter the Surface Charge

**FIGURE 2:**
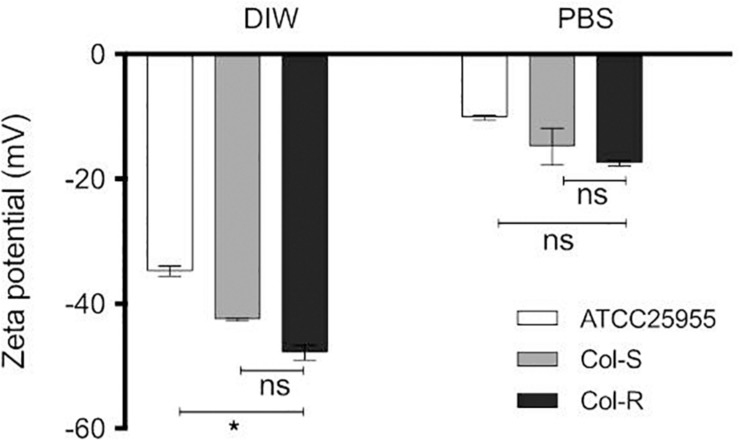
Distribution of the zeta potential values in two diluents: DIW and PBS. Zeta potential measurements were performed for Kpn strains (Col-R, Col-S, ATCC-25955) of 1 × 10^8^ CFU/ml in two different diluents DIW and PBS. There were no statistically significant differences between the Col-R and Col-S isolates (*P* = 0.54 in DIW and *P* ≥ 0.99 in PBS). However, there was a statistically significant difference between Col-R and ATCC strain in DIW (^∗^ in the figure, *P* = 0.022), but not in PBS (*P* = 0.30). Data plotted are mean ± SEM and represent the average of triplicates from three independent experiments. The Kruskal–Wallis test was used to compare the three bacterial strains. DIW; sterile deionized water. PBS; phosphate buffer saline.

To examine the effect of *mgrB*-insertion on bacterial surface charge, the zeta potential for three Kpn isolates was measured. All strains exhibited a negative surface charge in deionized water (DIW) and PBS (Figure 2). The zeta potential values for the cells dispersed in PBS were lower compared to the values obtained in DIW, probably due to the higher ionic strength in PBS that can suppress the surface charge of the bacteria (Table 2). Unexpectedly, there was no obvious difference between the Col-R and Col-S isolates with regard to surface charge (*P* = 0.54 in DIW and *P* ≥ 0.99 in PBS). Notably, the Col-R isolate had a more negative surface charge compared to the ATCC control isolate in water (*P* = 0.022, Dunn’s *post hoc* test), but not in PBS (*P* = 0.30, Figure 2).

**TABLE 2:** Zeta potential values of bacteria in two different solvents (PBS and DIW water).

**Bacterial strains**	**Bacterial count (CFU log_10_)**	**Zeta Potential (PBS) (Mean mV ± SD)**	**Zeta Potential (DIW) (Mean mV ± SD)**
ATCC 25955	7.8	−10.18 ± 0.59	−34.82 ± 1.44
Col-S (OM322)	7.6	−14.83 ± 5.03	−42.56 ± 0.41
Col-R (OM124)	7.6	−17.49 ± 0.73	−7.88 ± 2.04

*Average values of three separate measurements are presented. SD, standard deviation; DIW, De-ionized water; PBS, Phosphate-buffered saline; CFU, colony count unit.*

### LL-37 Causes Membrane-Damage and Intracellular Changes in Both Col-S and Col-R

**FIGURE 3:**
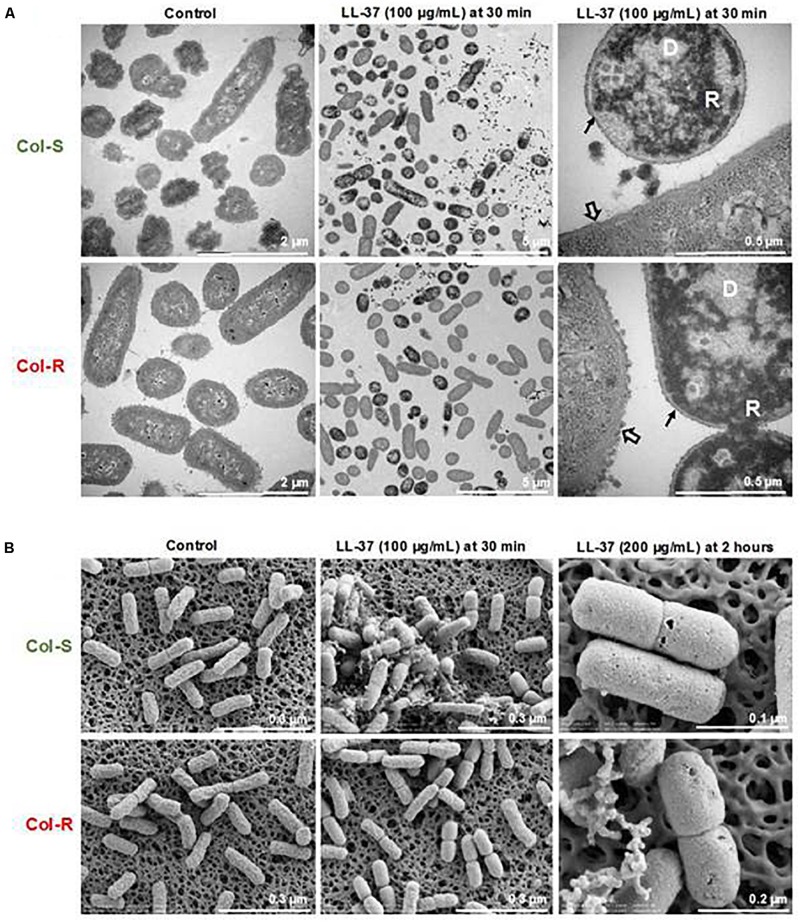
LL-37 induced morphological changes to both colistin-resistant and susceptible isolates. Representative images are shown for the intracellular changes detected by TEM **(A)** and surface morphological changes visualized by SEM **(B)**. Col-R and Col-S (1 × 10^8^ CFU/ml) were exposed to different concentrations of LL-37 (100 and 200 μg/ml) for 30 min, then visualized by TEM and SEM. One representative experiment out of three is shown. D; clustered DNA. R; clustered ribosomes. Empty arrow; capsule. Black arrow; loss of capsule.

Potential morphological changes in Kpn as a result of exposure to LL-37 were examined. To that end, intracellular morphological changes of bacterial cells after incubation with different concentrations of LL-37 were determined using TEM. Non-treated bacteria had intact membranes with an even distribution of DNA (light area) and ribosome-rich areas (dark area, Figure 3A). Bacterial exposure to LL-37 (100 μg/ml) for 30 min induced morphological changes inside the bacterial cells, such as clustering of DNA and ribosomes in both Col-R and Col-S strains. Treatment with LL-37 appeared to cause the loss of the capsule layer in both isolates, even though that the Col-R isolate was less susceptible to LL-37 concentrations above 50 μg/ml based on CFU-counts (Figure 3A and Supplementary Figure S2). SEM was performed to assess potential changes in the surface structure. Bacterial cells of Col-S and Col-R exposed to 100 μg/ml of LL-37 for 30 min appeared to be more swollen compared to untreated controls (Figure 3B). However, treatment with 100 and 200 μg/ml of LL-37 for 2 h caused swelling and pore formation in both strains (Figure 3B). Overall, LL-37 caused profound changes in both the Col-S and Col-R strains but no obvious differences between the isolates could be observed using TEM or SEM.

### No Difference in Pathogenicity or Virulence Between Colistin-Resistant and Susceptible Kpn-Isolates

**FIGURE 4:**
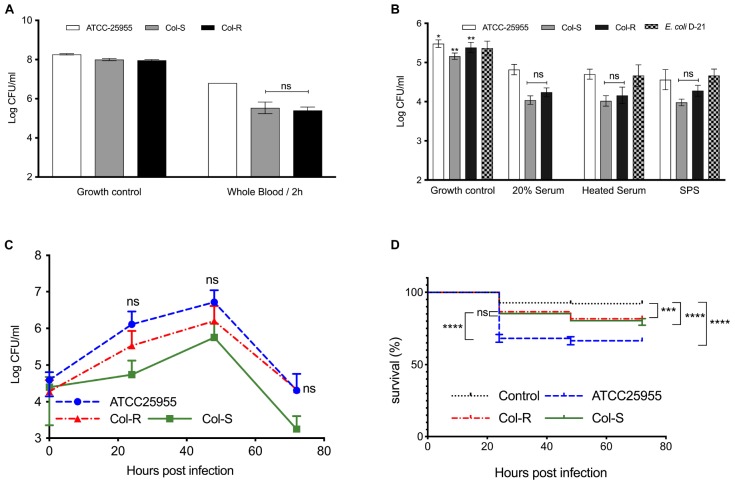
Pathogenicity and virulence of colistin-resistant and susceptible isolates. **(A)** Bacteria (∼5 × 10^7^ CFU/ml) were incubated in diluted human whole blood for 2 h. **(B)** Diluted sera obtained from healthy individuals were incubated for 2 h with bacteria (∼5 × 10^4^ CFU/ml) directly or after complement inactivation by heating or adding sodium polyanethole sulfonate (SPS). *E. coli* D-21, known to be susceptible to serum-killing, was used as a control. The lowest limit of detection was ∼1 × 10^2^ CFU. Data plotted are mean ± SEM and represent at least three independent experiments. n.s.; not significant; non-parametric Kruskal–Wallis test. **(C,D)** Zebrafish embryos were infected with 150–300 CFU of Col-R, Col-S, ATCC Kpn or medium, following incubation at 30°C for 72 h. **(C)** Bacterial growth in the infected embryos over time is shown. Whole embryos were lysed (*n* = 1–3) for CFU count. No statistically significant difference between Col-R and Col-S was observed. Data plotted are mean ± SD. The non-parametric Kruskal–Wallis test was used for statistical analysis. **(D)** Survival curves for infected and control embryos are shown. No statistically significant difference was observed between Col-R and Col-S strains, whereas a significant difference between embryos infected with the ATCC strain could be noted. Data plotted are mean ± SEM and represent four independent experiments. Log-rank (Mantel-Cox) test was used for survival analyses. ^∗∗∗^*P* ≤ 0.001; ^****^P ≤ 0.0001.

Since concentrations of LL-37 above 50 μg/ml can be considered to be supra-physiological, we tested the survival of colistin susceptible and resistant strains in whole blood and serum, which are complex biological fluids and representative of innate effector mechanisms in humans. In whole blood, the growth of Col-R and Col-S were reduced with more than 2 log_10_ within 2 h. In contrast, the Kpn control-strain (ATCC25955) was only reduced by 0.5 log_10_ (Figure 4A). We further investigated the potential cause of the killing in the clinical strains by testing bacterial growth in serum; using both an intact and inactivated complement system (Figure 4B). The control-strain *E. coli* D21 was readily cleared in 20% serum, but grew well when complement was inactivated. Notably, all three Kpn strains were slightly sensitive to 20% serum, however, the clinical strains (Col-R and Col-S) were more sensitive to serum compared to the Kpn control-strain (ATCC25955). Interestingly, the serum-mediated killing of Kpn appeared to be independent of complement, since inactivation of this system did not affect bacterial growth.

To obtain further support for our findings from human blood and serum, a zebrafish model was employed. All three isolates grew well in the zebrafish-embryos, with an increase of two log_10_ units up to 48 h post infection (Figure 4C). After 48 h, bacterial growth declined quite rapidly, which could be explained by the fact that fish innate immune cells, particularly neutrophils, develop after 32–48 h post fertilization (Fehr et al., 2015). The ATCC-strain reached slightly higher CFU-counts versus the Col-R and Col-S isolates, but the difference was not statistically significant. Notably, there was no significant difference between the Col-R and Col-S isolates with regard to embryo survival (Figure 4D).

## Discussion

In this study, we only observed cross resistance between colistin and LL-37 at concentrations above 50 μg/ml (or 11 μM) – but not below this concentration - for LL-37 in Kpn-isolates resistant to colistin due to an insertion element in the *mgrB* gene. Further analysis showed that the surface charge of the Col-R and Col-S isolates was similar. In addition, both isolates showed morphological alterations and membrane damage after exposure to LL-37, independent of colistin resistance. Finally, both colistin-resistant and colistin-susceptible strains were rapidly cleared by blood and serum, as well as in a zebrafish model.

Despite the fact that colistin and LL-37 exhibit similar binding mechanisms to LPS, the question of whether there is a cross-resistance between colistin and LL-37 is still unresolved. The potential cross-resistance is a worrying concern since AMPs are effectors of the innate immune system, and resistance could compromise the natural defenses against pathogens (Bell and Gouyon, 2003). Previous data on cross resistance between colistin and LL-37 are not conclusive. For example, in *A*. *baumannii* there are several reports regarding cross-resistance (negative, positive and null effects) (Moffatt et al., 2013; Napier et al., 2013; Garcia-Quintanilla et al., 2014). For Kpn, no cross-resistance between colistin and LL-37 has been observed, in either isolates with *mgrB* inactivation due to IS5-like insertions or in isolates with other chromosomal mechanisms (Dobias et al., 2017). Notably, in the report by Dobias et al. (2017) the effects of LL-37 were assessed at a lower range than the one used in this study (<20 μg/ml or <4.5 μM). However, Kadar et al. (2015) reported cross-resistance in Kpn between colistin and other antibacterial compounds, including lactoferrin, lysozyme, and protamine. Additionally Llobet et al. (2009) reported cross resistance with human α-defensin 1 (HNP-1). It should be noted, however, that there is no consensus on how to perform these assays and the methodology differs significantly between laboratories. For example, the protocols differed with regard to media-composition, incubation time, and initial bacterial inoculum, and each study has included unique strains with different resistance mechanisms.

The physiological level of LL-37 varies within different tissues and cells. Sorensen et al., determined the mean levels of hCAP18 (the pro-form of LL-37) in plasma to be 1.18 μg/ml (0.07 μM) and in serum as 0.93 μg/ml (0.06 μM). While in neutrophils they reported hCAP18 level to be as high as 640 μg/ml (40 μM) in normal healthy volunteers (Sorensen et al., 1997). In normal human skin, LL-37 is synthesized by epithelial cells and the levels are usually low (Frohm et al., 1997). However, during pathological conditions the levels of LL-37 have been found to be significantly higher. In multiple trauma patients, LL-37 levels were induced 15-fold in plasma (Lippross et al., 2012). In addition, during skin infection or injury, the production is strongly induced (Frohm et al., 1997; Dorschner et al., 2001; Schauber et al., 2007). Furthermore, the level of LL-37 was reported to be high in skin lesions due to inflammatory diseases, such as psoriasis or rosacea (Reinholz et al., 2012). Moreover, LL-37 levels were significantly elevated in serum specimens from psoriasis patients to as high level as 1366 μg/ml (304 μM) (Ong et al., 2002).

Given these data and our findings, we can conclude that colistin-resistant strains would be unaffected in plasma or serum from healthy individuals, where LL-37 is present at low concentrations. However, during certain conditions a higher release of LL-37 by neutrophils at the site of infection might give an advantage for resistant strains to survive, compared to colistin-susceptible strains. A similar selection pressure could be expected in diseases with elevated levels of LL-37, such as psoriasis, although this hypothesis remains to be proven.

Resistance to colistin is considered to be caused by a reduced net negative charge of the LPS molecule with amine substituents (L-4Ara4N and pEtN), which reduces the binding affinity of colistin (Choi et al., 2016). A reduced surface charge has been observed in strains that are naturally resistant to colistin, e.g., *P. mirabilis* (Sud and Feingold, 1970). The modification is achieved by a large panel of genes mostly found on chromosomes including, but not limited to, *mgrB*. Hence, *mgrB* alteration plays a prominent role in colistin resistance (Olaitan et al., 2014). We hypothesized that Col-R isolates would have lower electronegative surface charge compared to Col-S based on the assumption that the *mgrB* mutation could be associated with the addition of Ara4N to lipid A (Cannatelli et al., 2014; Wright et al., 2015). However, we did not observe statistically significant differences between Col-S and Col-R isolates in either water or PBS (Figure 2). Our finding is consistent with Ayerbe-Algaba et al. (2018) who reported a minor difference of zeta potential values in water for two clinical Kpn-isolates, Col-S and Col-R of −36.7 ± 0.88 and −35.27 ± 0.72 mV, respectively. The Col-R was due to *mgrB*-insertion, as was the case with the strains we studied here (Ayerbe-Algaba et al., 2018). In contrast, Velkov et al. (2014) reported that the zeta potentials for Col-S were significantly lower than Col-R isolates in mid-logarithmic growth phase in water. Taken together, our data suggest that the surface charge is not a major determinant of colistin-resistance in Kpn.

Apart from the proposed lipid A dephosphorylation, other factors could be controlled by *mgrB* gene. For example, deacylation of lipid A has been described, which does not appear to alter the surface charge but reduces susceptibility to AMPs (Clements et al., 2007). Thus, colistin-resistance caused by *mgrB* insertions could be related to changes in hydrophobic interactions or loss of the O-specific side chains in LPS, as in *Bordetella bronchiseptica* (Banemann et al., 1998), which is consistent with the fact that the strains studied here had different O serotypes (Table 1). Strain OM124 had O3b while strain OM322 had OL104 O-serotypes. OL104 is closely related to O3 serotypes (Follador et al., 2016). However, as we and others (Iwahi et al., 1982; Sahly et al., 2004) report, the O-serotype appears not to impact the susceptibility to serum. In all, factors implicated in the transport of peptides across the outer membrane are important for peptide resistance in Gram-negative bacteria. These factors are not limited to the charge of the LPS molecules, and could include the O-antigen (Weiss et al., 1980; Rana et al., 1991).

To investigate whether a fitness cost could be observed for the bacteria, we tested the isolates against other host defense mechanisms, using whole blood and serum. Importantly, both the Col-S and Col-R isolates were rapidly killed in whole blood and serum. Combined, these results suggest that the *mgrB*-insertion did not impact the survival in human blood or serum. Moreover, our results indicate that MDR Kpn-isolates are more susceptible to human blood and serum compared to a control strain. Additionally, data from the zebrafish model showed that the control strain reached higher bacterial counts and caused higher mortality compared to Col-R and Col-S, which could indicate a lower fitness of these isolates, consistent with their MDR-phenotype. However, it should be noted that the control isolate was different from the MDR Kpn used in this study with regard to genetic background and sequence type. Thus, conclusions regarding potential fitness-costs of MDR Kpn require additional studies. In addition, the data from the zebrafish embryo-model showed that there was no difference between Col-R and Col-S with regard to bacterial growth or survival, suggesting that *mgrB*-insertions do not impact bacterial fitness in relation to innate immunity in a primitive model system, like the zebrafish.

The question of whether *mgrB*-insertion and the Col-R phenotype has any impact on virulence has been addressed in previous studies. Others reported that there was no cost of fitness associated with colistin resistance due to *mgrB*-insertion *in vitro* as well as *in vivo* using the invertebrate *G. mellonella* infection model (Cannatelli et al., 2015; Arena et al., 2016). In contrast, Kidd et al. (2017), showed that *mgrB*-inactivation increased virulence in the same infection model (*G. mellonella*), yet no effect was observed in a mouse model of pneumonia. Thus, the role of *mgrB*-insertions and the Col-R phenotype for virulence *in vivo* has still not been fully resolved.

In conclusion, we found cross-resistance between colistin and LL-37 at concentrations above 50 μg/ml in Kpn-isolates with colistin-resistance due to *mgrB*-insertions but not below this concentration. There was no difference between the Col-R and Col-S isolates with regard to survival in human serum or whole blood, or in a zebrafish infection-model. The clinical implications of these findings are unclear, but it is possible that cross-resistance between colistin and LL-37 can play a role during infection or inflammation, where LL-37 concentrations may be high enough to exert a selection pressure on bacterial growth. It should be noted that potential interactions between antimicrobial resistant bacteria and innate effector systems remain largely unexplored. A detailed understanding of this area could open up for novel therapeutic options against multidrug-resistant bacteria.

## Data Availability Statement

The datasets generated for this study can be found in the Raw reads data generated in this study were deposited in SRA as project COL-R SAMN (11853672-80) and COL-S SAMN (11853647-54).

## Ethics Statement

Ethical approval was obtained from the Ministry of Health in Oman for the use of the strains and the collection of (anonymized) patient data (MH/DG/R&S/32/2015). The procedure to draw blood from healthy volunteers was approved by the Regional Ethical Review Board in Stockholm (dnr 2000-360/00). The patients/participants provided their written informed consent to participate in this study. The zebrafish study was approved by the Ethical Review Board Stockholm Animal Research Committee (dnr 19204-2017) and by the Swedish Board of Agriculture. Written informed consent was obtained from the individual(s) for the publication of any potentially identifiable images or data included in this article.

## Author Contributions

All authors listed have made a substantial, direct and intellectual contribution to the work, and approved it for publication.

## Conflict of Interest

The authors declare that the research was conducted in the absence of any commercial or financial relationships that could be construed as a potential conflict of interest.

## References

[B1] Arena F. Henrici De Angelis L. Cannatelli A. Di Pilato V. Amorese M. D’andrea M. M. (2016). Colistin resistance caused by inactivation of the MgrB regulator is not associated with decreased virulence of sequence type 258 KPC carbapenemase-producing *Klebsiella pneumoniae*. *Antimicrob. Agents Chemother.* 60 2509–2512. 10.1128/AAC.02981-15 26824959 PMC4808163

[B2] Ayerbe-Algaba R. Gil-Marques M. L. Jimenez-Mejias M. E. Sanchez-Encinales V. Parra-Millan R. Pachon-Ibanez M. E. (2018). Synergistic activity of niclosamide in combination with colistin against colistin-susceptible and colistin-resistant *Acinetobacter baumannii* and *Klebsiella pneumoniae*. *Front. Cell. Infect. Microbiol.* 8:348. 10.3389/fcimb.2018.00348 30338245 PMC6178895

[B3] Banemann A. Deppisch H. Gross R. (1998). The lipopolysaccharide of Bordetella bronchiseptica acts as a protective shield against antimicrobial peptides. *Infect. Immun.* 66 5607–5612. 9826332 10.1128/iai.66.12.5607-5612.1998 PMC108708

[B4] Bankevich A. Nurk S. Antipov D. Gurevich A. A. Dvorkin M. Kulikov A. S. (2012). SPAdes: a new genome assembly algorithm and its applications to single-cell sequencing. *J. Comput. Biol.* 19 455–477. 10.1089/cmb.2012.0021 22506599 PMC3342519

[B5] Bell G. Gouyon P. H. (2003). Arming the enemy: the evolution of resistance to self-proteins. *Microbiology* 149 1367–1375. 10.1099/mic.0.26265-0 12777478

[B6] Cannatelli A. Giani T. D’andrea M. M. Di Pilato V. Arena F. Conte V. (2014). MgrB inactivation is a common mechanism of colistin resistance in KPC-producing *Klebsiella pneumoniae* of clinical origin. *Antimicrob. Agents Chemother.* 58 5696–5703. 10.1128/AAC.03110-14 25022583 PMC4187966

[B7] Cannatelli A. Santos-Lopez A. Giani T. Gonzalez-Zorn B. Rossolini G. M. (2015). Polymyxin resistance caused by mgrB inactivation is not associated with significant biological cost in *Klebsiella pneumoniae*. *Antimicrob. Agents Chemother.* 59 2898–2900. 10.1128/AAC.04998-14 25691629 PMC4394794

[B8] Choi M. J. Kim S. Ko K. S. (2016). Pathways regulating the pbgP operon and colistin resistance in *Klebsiella pneumoniae* Strains. *J. Microbiol. Biotechnol.* 26 1620–1628. 10.4014/jmb.1604.04016 27238937

[B9] Clements A. Tull D. Jenney A. W. Farn J. L. Kim S. H. Bishop R. E. (2007). Secondary acylation of *Klebsiella pneumoniae* lipopolysaccharide contributes to sensitivity to antibacterial peptides. *J. Biol. Chem.* 282 15569–15577. 10.1074/jbc.m701454200 17371870 PMC5007121

[B10] Dobias J. Poirel L. Nordmann P. (2017). Cross-resistance to human cationic antimicrobial peptides and to polymyxins mediated by the plasmid-encoded MCR-1? *Clin. Microbiol. Infect.* 23 e671–e676. 10.1016/j.cmi.2017.03.015 28344161

[B11] Dorschner R. A. Pestonjamasp V. K. Tamakuwala S. Ohtake T. Rudisill J. Nizet V. (2001). Cutaneous injury induces the release of cathelicidin anti-microbial peptides active against group A Streptococcus. *J. Invest. Dermatol.* 117 91–97. 10.1046/j.1523-1747.2001.01340.x 11442754

[B12] Fehr A. Eshwar A. K. Neuhauss S. C. Ruetten M. Lehner A. Vaughan L. (2015). Evaluation of zebrafish as a model to study the pathogenesis of the opportunistic pathogen *Cronobacter turicensis*. *Emerg. Microbes Infect.* 4:e29. 10.1038/emi.2015.29 26060602 PMC4451267

[B13] Follador R. Heinz E. Wyres K. L. Ellington M. J. Kowarik M. Holt K. E. (2016). The diversity of *Klebsiella pneumoniae* surface polysaccharides. *Microb. Genomics* 2:e000073. 10.1099/mgen.0.000073 28348868 PMC5320592

[B14] Frohm M. Agerberth B. Ahangari G. Stahle-Backdahl M. Liden S. Wigzell H. (1997). The expression of the gene coding for the antibacterial peptide LL-37 is induced in human keratinocytes during inflammatory disorders. *J. Biol. Chem.* 272 15258–15263. 10.1074/jbc.272.24.15258 9182550

[B15] Fukuoka S. Howe J. Andra J. Gutsmann T. Rossle M. Brandenburg K. (2008). Physico-chemical and biophysical study of the interaction of hexa- and heptaacyl lipid A from Erwinia carotovora with magainin 2-derived antimicrobial peptides. *Biochim. Biophys. Acta* 1778 2051–2057. 10.1016/j.bbamem.2008.03.022 18440300

[B16] Garcia-Quintanilla M. Pulido M. R. Moreno-Martinez P. Martin-Pena R. Lopez-Rojas R. Pachon J. (2014). Activity of host antimicrobials against multidrug-resistant *Acinetobacter baumannii* acquiring colistin resistance through loss of lipopolysaccharide. *Antimicrob. Agents Chemother.* 58 2972–2975. 10.1128/AAC.02642-13 24566189 PMC3993257

[B17] Giske C. G. (2015). Contemporary resistance trends and mechanisms for the old antibiotics colistin, temocillin, fosfomycin, mecillinam and nitrofurantoin. *Clin. Microbiol. Infect* 21 899–905. 10.1016/j.cmi.2015.05.022 26027916

[B18] Iwahi T. Abe Y. Tsuchiya K. (1982). Virulence of *Escherichia coli* in ascending urinary-tract infection in mice. *J. Med. Microbiol.* 15 303–316. 10.1099/00222615-15-3-303 6126593

[B19] Jolley K. A. Maiden M. C. (2010). BIGSdb: scalable analysis of bacterial genome variation at the population level. *BMC Bioinformatics* 11:595. 10.1186/1471-2105-11-595 21143983 PMC3004885

[B20] Kadar B. Kocsis B. Kristof K. Toth A. Szabo D. (2015). Effect of antimicrobial peptides on colistin-susceptible and colistin-resistant strains of *Klebsiella pneumoniae* and *Enterobacter asburiae*. *Acta Microbiol. Immunol. Hung.* 62 501–508. 10.1556/030.62.2015.4.12 26689883

[B21] Kidd T. J. Mills G. Sa-Pessoa J. Dumigan A. Frank C. G. Insua J. L. (2017). A *Klebsiella pneumoniae* antibiotic resistance mechanism that subdues host defences and promotes virulence. *EMBO Mol. Med.* 9 430–447. 10.15252/emmm.201607336 28202493 PMC5376759

[B22] Lippross S. Klueter T. Steubesand N. Oestern S. Mentlein R. Hildebrandt F. (2012). Multiple trauma induces serum production of host defence peptides. *Injury* 43 137–142. 10.1016/j.injury.2011.03.044 21561617

[B23] Liu Y.-Y. Wang Y. Walsh T. R. Yi L.-X. Zhang R. Spencer J. (2016). Emergence of plasmid-mediated colistin resistance mechanism MCR-1 in animals and human beings in China: a microbiological and molecular biological study. *Lancet Infect. Dis.* 16 161–168. 10.1016/S1473-3099(15)00424-7 26603172

[B24] Llobet E. March C. Gimenez P. Bengoechea J. A. (2009). *Klebsiella pneumoniae* OmpA confers resistance to antimicrobial peptides. *Antimicrob. Agents Chemother.* 53 298–302. 10.1128/AAC.00657-08 19015361 PMC2612193

[B25] Marcoleta A. E. Varas M. A. Ortiz-Severin J. Vasquez L. Berrios-Pasten C. Sabag A. V. (2018). Evaluating different virulence traits of *Klebsiella pneumoniae* using dictyostelium discoideum and zebrafish larvae as host models. *Front. Cell. Infect. Microbiol.* 8:30. 10.3389/fcimb.2018.00030 29479519 PMC5811510

[B26] McArthur A. G. Waglechner N. Nizam F. Yan A. Azad M. A. Baylay A. J. (2013). The comprehensive antibiotic resistance database. *Antimicrob. Agents Chemother.* 57 3348–3357. 10.1128/AAC.00419-13 23650175 PMC3697360

[B27] Meier-Kolthoff J. P. Auch A. F. Klenk H.-P. Göker M. (2013). Genome sequence-based species delimitation with confidence intervals and improved distance functions. *BMC Bioinformatics* 14:60. 10.1186/1471-2105-14-60 23432962 PMC3665452

[B28] Moffatt J. H. Harper M. Mansell A. Crane B. Fitzsimons T. C. Nation R. L. (2013). Lipopolysaccharide-deficient *Acinetobacter baumannii* shows altered signaling through host Toll-like receptors and increased susceptibility to the host antimicrobial peptide LL-37. *Infect. Immun.* 81 684–689. 10.1128/IAI.01362-12 23250952 PMC3584870

[B29] Mohsin J. Pal T. Petersen J. E. Darwish D. Ghazawi A. Ashraf T. (2018). Plasmid-Mediated colistin resistance gene mcr-1 in an *Escherichia coli* ST10 bloodstream isolate in the sultanate of Oman. *Microb. Drug Resist.* 24 278–282. 10.1089/mdr.2017.0131 28799833

[B30] Napier B. A. Burd E. M. Satola S. W. Cagle S. M. Ray S. M. Mcgann P. (2013). Clinical use of colistin induces cross-resistance to host antimicrobials in *Acinetobacter baumannii*. *mBio* 4:e21-13. 10.1128/mBio.00021-13 23695834 PMC3663567

[B31] Olaitan A. O. Morand S. Rolain J. M. (2014). Mechanisms of polymyxin resistance: acquired and intrinsic resistance in bacteria. *Front. Microbiol.* 5:643. 10.3389/fmicb.2014.00643 25505462 PMC4244539

[B32] Ong P. Y. Ohtake T. Brandt C. Strickland I. Boguniewicz M. Ganz T. (2002). Endogenous antimicrobial peptides and skin infections in atopic dermatitis. *N. Engl. J. Med.* 347 1151–1160. 10.1056/nejmoa021481 12374875

[B33] Paczosa M. K. Mecsas J. (2016). *Klebsiella pneumoniae*: going on the offense with a strong defense. *Microbiol. Mol. Biol. Rev.* 80 629–661. 10.1128/MMBR.00078-15 27307579 PMC4981674

[B34] Palarasah Y. Skjoedt M.-O. Vitved L. Andersen T. E. Skjoedt K. Koch C. (2010). Sodium polyanethole sulfonate as an inhibitor of activation of complement function in blood culture systems. *J. Clin. Microbiol.* 48 908–914. 10.1128/JCM.01985-09 20042630 PMC2832435

[B35] Rana F. R. Macias E. A. Sultany C. M. Modzrakowski M. C. Blazyk J. (1991). Interactions between magainin 2 and *Salmonella typhimurium* outer membranes: effect of lipopolysaccharide structure. *Biochemistry* 30 5858–5866. 10.1021/bi00238a008 2043628

[B36] Reinholz M. Ruzicka T. Schauber J. (2012). Cathelicidin LL-37: an antimicrobial peptide with a role in inflammatory skin disease. *Ann. Dermatol.* 24 126–135. 10.5021/ad.2012.24.2.126 22577261 PMC3346901

[B37] Rodriguez-R L. M. Konstantinidis K. T. (2016). The enveomics collection: a toolbox for specialized analyses of microbial genomes and metagenomes. *PeerJ Preprints* 4:e1900–e1901.

[B38] Sahly H. Aucken H. Benedí V. J. Forestier C. Fussing V. Hansen D. S. (2004). Increased serum resistance in *Klebsiella pneumoniae* strains producing extended-spectrum beta-lactamases. *Antimicrob. Agents Chemother.* 48 3477–3482. 10.1128/aac.48.9.3477-3482.2004 15328114 PMC514775

[B39] Schauber J. Dorschner R. A. Coda A. B. Buchau A. S. Liu P. T. Kiken D. (2007). Injury enhances TLR2 function and antimicrobial peptide expression through a vitamin D-dependent mechanism. *J. Clin. Invest.* 117 803–811. 10.1172/jci30142 17290304 PMC1784003

[B40] Siguier P. Perochon J. Lestrade L. Mahillon J. Chandler M. (2006). ISfinder: the reference centre for bacterial insertion sequences. *Nucleic Acids Res.* 34 D32–D36. 16381877 10.1093/nar/gkj014 PMC1347377

[B41] Sonnevend A. Ghazawi A. Alqahtani M. Shibl A. Jamal W. Hashmey R. (2016). Plasmid-mediated colistin resistance in *Escherichia coli* from the Arabian Peninsula. *Int. J. Infect. Dis.* 50 85–90. 10.1016/j.ijid.2016.07.007 27566913

[B42] Soon R. L. Nation R. L. Cockram S. Moffatt J. H. Harper M. Adler B. (2011). Different surface charge of colistin-susceptible and -resistant *Acinetobacter baumannii* cells measured with zeta potential as a function of growth phase and colistin treatment. *J. Antimicrob. Chemother.* 66 126–133. 10.1093/jac/dkq422 21081544 PMC3001852

[B43] Sorensen O. Cowland J. B. Askaa J. Borregaard N. (1997). An ELISA for hCAP-18, the cathelicidin present in human neutrophils and plasma. *J. Immunol. Methods* 206 53–59. 10.1016/s0022-1759(97)00084-7 9328568

[B44] Sud I. J. Feingold D. S. (1970). Mechanism of polymyxin B resistance in *Proteus mirabilis*. *J. Bacteriol.* 104 289–294. 4319722 10.1128/jb.104.1.289-294.1970 PMC248213

[B45] Sun J. Zhang H. Liu Y. H. Feng Y. (2018). Towards understanding MCR-like colistin resistance. *Trends Microbiol* 26 794–808. 10.1016/j.tim.2018.02.006 29525421

[B46] The European Committee on Antimicrobial Susceptibility Testing [EUCAST] (2016). *Breakpoint Tables for Interpretation of MICs and Zone Diameters.* Basel: EUCAST.

[B47] Velkov T. Deris Z. Z. Huang J. X. Azad M. A. Butler M. Sivanesan S. (2014). Surface changes and polymyxin interactions with a resistant strain of *Klebsiella pneumoniae*. *Innate Immun* 20 350–363. 10.1177/1753425913493337 23887184 PMC4242413

[B48] Weiss J. Beckerdite-Quagliata S. Elsbach P. (1980). Resistance of gram-negative bacteria to purified bactericidal leukocyte proteins: relation to binding and bacterial lipopolysaccharide structure. *J. Clin. Invest.* 65 619–628. 10.1172/jci109707 6986410 PMC371403

[B49] Wick R. R. Heinz E. Holt K. E. Wyres K. L. (2018). Kaptive web: user-friendly capsule and lipopolysaccharide serotype prediction for *Klebsiella* genomes. *J. Clin. Microbiol.* 56:e19718. 10.1128/JCM.00197-18 29618504 PMC5971559

[B50] Wright M. S. Suzuki Y. Jones M. B. Marshall S. H. Rudin S. D. Van Duin D. (2015). Genomic and transcriptomic analyses of colistin-resistant clinical isolates of *Klebsiella pneumoniae* reveal multiple pathways of resistance. *Antimicrob. Agents Chemother.* 59 536–543. 10.1128/AAC.04037-14 25385117 PMC4291396

[B51] Zankari E. Hasman H. Cosentino S. Vestergaard M. Rasmussen S. Lund O. (2012). Identification of acquired antimicrobial resistance genes. *J Antimicrob Chemother* 67 2640–2644. 10.1093/jac/dks261 22782487 PMC3468078

[B52] Zhou Y. Wang J. Guo Y. Liu X. Liu S. Niu X. (2019). Discovery of a potential MCR-1 inhibitor that reverses polymyxin activity against clinical mcr-1-positive *Enterobacteriaceae*. *J. Infect.* 78 364–372. 10.1016/j.jinf.2019.03.004 30851289

