# Supplementary material for: Effects of the Antimicrobial Peptide LL-37 and Innate Effector Mechanisms in Colistin-Resistant Klebsiella pneumoniae With mgrB Insertions
Source: Front Microbiol. 2019 Nov 14;10:2632. doi: 10.3389/fmicb.2019.02632 (PMC6870453; doi:10.3389/fmicb.2019.02632)
Supplement: Supplementary file 1 [file Data_Sheet_1.pdf]

Supplementary Material

1 Supplementary Data

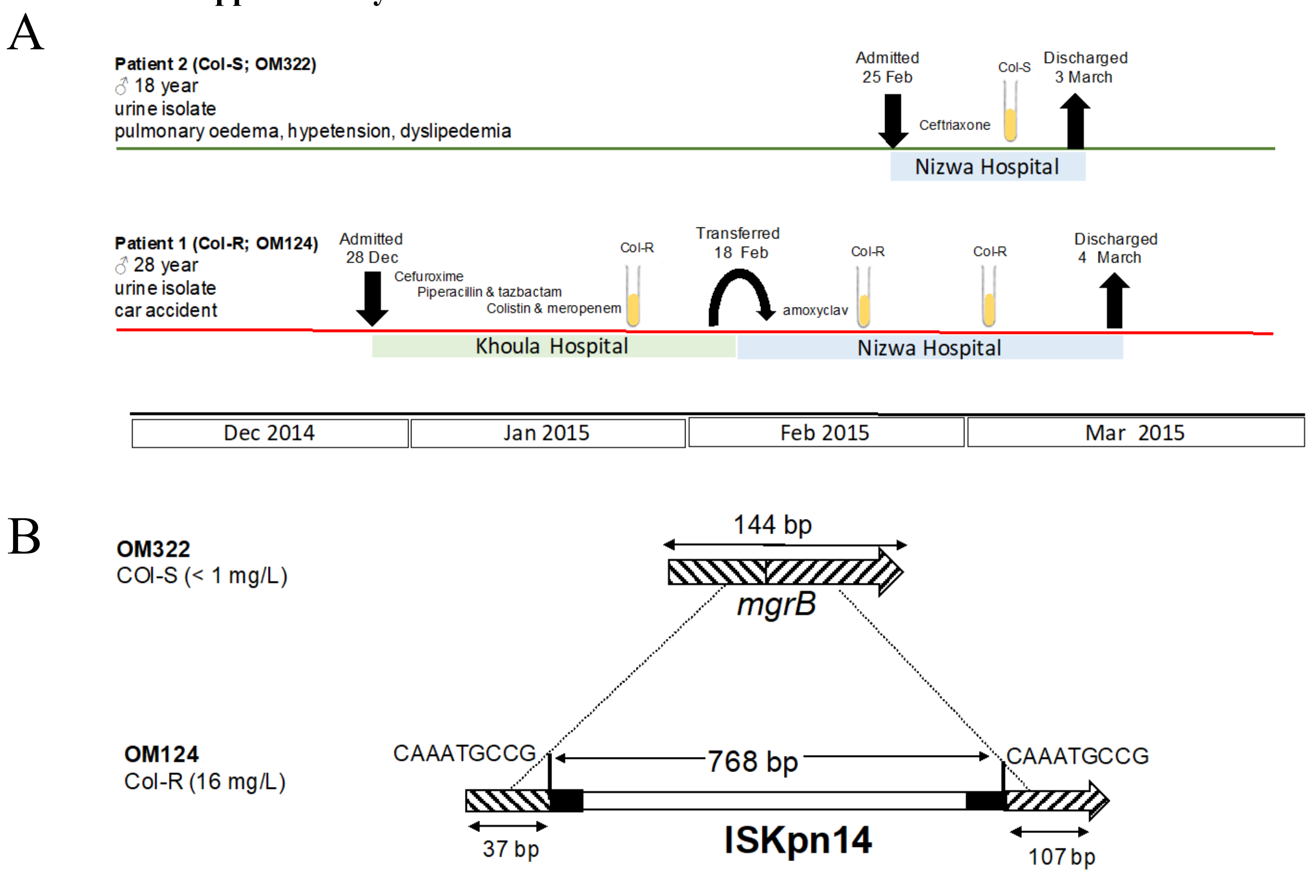

Fig. S1. Schematic representation of clinical and gentic charcteristics of the genetically similar isolates Col-S and Col-R. (A) A colistin resistant strain (OM124) was isolated from a 28-year old male patient admitted to the neuro-surgery ward at Khoula hospital, Oman, after a road traffic accident, which resulted in a severe head injury on December 28, 2014. The patient initially received cefuroxime, then piperacillin-tazobactam, and finally was treated with a combination of colistin and meropenem. He was transferred to Nizwa hospital, Oman, on February 18, 2015, where he was admitted to the general surgery ward for one month. Urine was cultured twice and showed a similar resistance pattern. He was discharged on March 4, 2015. From the transferring hospital, Nizwa, the colistin susceptible strain (OM322) was isolated from an 18-year old male admitted to the ICU on February 25, 2015 and discharged on March 3, 2015. The patient was treated with ceftriaxone. We speculate that the colistin-resistant strain reverted to colistin-susceptibility when colistin exposure was ceased. (B) The sight of integration of insertion element (IS) in *mgrB* locus of colistin-resistant and colistin-susceptible *K. pneumoniae*. IS is shown as rectangle with inverted repeats (IRs) depicted in black. The interrupted *mgrB* fragments are shown with crossed lines. The DNA sequences of the short directed repeat (DR) generated by transposition are shown. ISKpn14 belongs to IS1 superfamily. Minimum Inhibitory Concentration (MIC) value for colistin shown in bracket. Col-R; colistin-resistant. Col-S; colistin-susceptible.

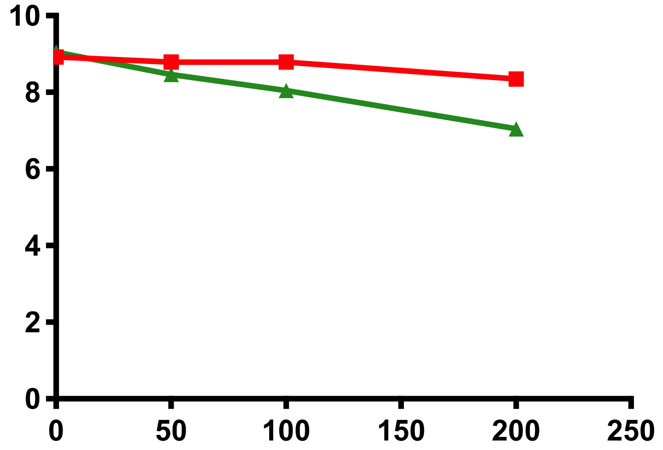

B

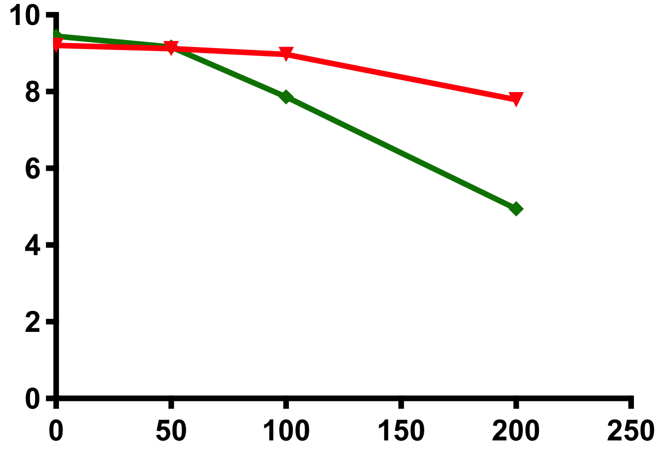

Fig. S2. For Transmission Electron Microscopy (TEM), Col-R and Col-S were used in a higher concentration, (1×10^8 CFU/ml). Both strains were incubated with different concentrations of LL-37 (50, 100 and 200 µg/ml) for 30 minutes (A) and 2 hours (B) at 37°C. Next, 10-fold dilutions were made, of wich 20 µl of the dilutions was spread on blood agar plates and incubated overnight at 37°C. On the next day colonies were counted to determine the number of surviving bacteria.
